# Why the development of effective typhoid control measures requires the use of human challenge studies

**DOI:** 10.3389/fmicb.2014.00707

**Published:** 2014-12-16

**Authors:** Claire Jones, Thomas C. Darton, Andrew J. Pollard

**Affiliations:** Oxford Vaccine Group, Department of Paediatrics, National Institute for Health Research Oxford Biomedical Research Centre, University of OxfordOxford, UK

**Keywords:** *Salmonella* Typhi, typhoid, enteric fever, human challenge studies, host-pathogen interactions, typhoid vaccine, diagnostics

## Introduction

*Salmonella* Typhi (*S*. Typhi) has affected mankind for the last 50,000 years (Kidgell et al., 2002; Roumagnac et al., 2006), however the precise pathogenesis in humans has largely remained a mystery (Crump and Mintz, 2010). Typhoid fever, the systemic disease caused by *S*. Typhi infection, is responsible for an estimated 21 million new infections annually resulting in approximately 200,000–600,000 deaths world-wide (Crump et al., 2004; Buckle et al., 2012). If untreated, typhoid fever may result in severe illness including the complications of gastrointestinal bleeding, bowel perforation, and sometimes death. Transmission of *S*. Typhi occurs via ingestion of faecally-contaminated food or water (De Jong et al., 2012). Infection risk has been associated with household factors including contact with a recently infected relative, poor sanitation and hygiene infrastructure, which include spatial associations with contaminated public water sources (Vollaard et al., 2004; Sur et al., 2009; Baker et al., 2011).

Much of what we know about human *Salmonella* infection has been determined from historical human vaccine and, subsequently, challenge studies dating back to 1896 (Waddington et al., 2014a). Between 1952 and 1974, human challenge studies performed at the University of Maryland served as a unique tool by which to study host-pathogen interactions including mechanistic hypotheses regarding routes of infection, development of clinical symptomatology and evolution of host immune responses.

Initial improvements in sanitation infrastructure occurring during the last century in “western settings” and subsequently in the rest of the world resulted in a general reduction in the prevalence of typhoid fever. Infection is still highly prevalent in resource-limited countries and travelers. Reasons for this include the lack of effective vaccine campaigns, availability of accurate diagnostic tests and the emergence of antibiotic resistance, hindered by the incomplete understanding of bacterial pathogenesis and response to infection by the native host.

To address this, in 2011 we re-established controlled human infection studies of *S*. Typhi in Oxford. A model was developed using a sodium bicarbonate buffer to neutralize gastric acid and increase bacterial survival through the stomach; neutralization of stomach acid allows the use of lower challenge inocula and a smoother pattern of clinical infection (Waddington et al., 2014b). Ingestion with buffer resulted in a consistent pattern of typhoid infection with an attack rate of 65%, which developed after ingestion of 1–5 × 10^4^ colony forming units. Participants were managed after challenge on an outpatient basis.

The development of this new challenge model will provide a standardized approach to study typhoid infection that will prove fundamental for the investigation of immunobiology in the relevant human host and the discovery, development and evaluation of novel vaccines, diagnostics and treatment modalities.

## Typhoid fever, a global problem

Typhoid fever is an important health problem in resource-limited settings, while its profile on the world stage is increasing due to the risk of infection for travelers (Leder et al., 2013). Approximately 80% of typhoid fever in Europe and North America is associated with travel, with the greatest proportion resulting from travel to the Indian subcontinent (Hendel-Paterson and Swanson, 2011). Vaccination is recommended by the World Health Organization (WHO) for all travelers to countries where enteric fever is endemic, regardless of the planned duration of stay in a typhoid-endemic setting. Although licensed vaccines provide protection against *S*. Typhi and the live oral vaccine Ty21a is thought to offer limited cross-protection against *S*. Paratyphi B infection, there is currently no licensed bivalent vaccine for *S*. Typhi and *S*. Paratyphi A, the leading causes of enteric fever (Pakkanen et al., 2012). Insights into the dynamics of host-pathogen interactions are crucial to understanding how *S*. Typhi exploits host defenses during infection and is able to occupy its human-restricted niche. Challenge studies could be described as the ideal at-risk travelers model, where naive adult participants are given a bacterial inoculum sufficient to cause typhoid fever. The model can be used to assess the potential of prophylactic vaccination to prevent infection using typhoid challenge and to accelerate the route to licensure, and field testing potential of innovative diagnostics.

Increasing antimicrobial resistance and the evolution of multi drug resistant strains reduces treatment options in many endemic settings (Dutta et al., 2014; Walters et al., 2014). Chloramphenicol, ampicillin and co-trimoxazole resistance emerged throughout the Indian subcontinent and South East Asia during the late 1980s (Holt et al., 2012). The isolation of quinolone resistant *S*. Typhi has become a cause for concern as drug resistance patterns make treatment options more difficult and costly. Diagnostics at the point of care are important for the prompt detection of causative bacteria, early appropriate case management and targeted antibiotic treatment (Laxminarayan et al., 2013). Large, well designed trials to determine optimal adult and pediatric treatment policies to prevent clinical relapse of enteric fever in endemic areas are important for the management of anti-microbial resistant *S*. Typhi and its associated public health burden (Thaver et al., 2009; Arjyal et al., 2011). In our own challenge programme, there is an opportunity to use the model as a platform to evaluate antibiotic treatment regimens and inform on pharmacodynamics in the resolution of infection.

Prevention of *S*. Typhi infection among travelers through pre-travel vaccination is fundamental as international travel becomes more accessible and a significant cause of travel-associated illness (Freedman et al., 2014). Government and public health authorities are becoming more involved in promoting immunization, personal hygiene education as well as implementing GeoSentinal sites to collect surveillance data including patient demographic characteristics, detailed travel history, vaccination status, and specific typhoid fever disease diagnosis (Leder et al., 2013). Practical applications for human challenge models to characterize infection rate, symptom severity, duration of shedding and biomarkers of disease are important for the identification of disease and mechanisms of protective immunity. Furthermore, the model may provide important supportive data for licensure of new travel vaccines for enteric fever.

## Why are human challenge studies so unique and what can we learn?

Most human *Salmonella* infections result in gastroenteritis and are caused by *Salmonella* Typhimurium or *Salmonella* Enteritidis. *S*. Typhi, the predominant etiologic agent of typhoid (enteric) fever, is specifically adapted to only infect humans with no known animal or environmental reservoir. Animal models for invasive non-typhoidal *Salmonella* (NTS) are available, an important source of human disease in the immunocompromised host (Simon et al., 2011). Despite the discovery and establishment of systemic (intravenous or intraperitoneal) and oral *S*. Typhi murine infection models in humanized and knockout mice (Levine et al., 2001; Andrews-Polymenis et al., 2010; Song et al., 2010; Mathur et al., 2012), the limited understanding of human infection has significantly hampered the development of new vaccines. The availability of tractable animal models of *S*. Typhi infection is of significant scientific interest but it must be argued that improved understanding and assessment of *S*. Typhi host-pathogen interactions can only effectively be achieved through the study of its natural host. The *S*. Typhi human challenge model is pivotal in addressing broad themes of basic *Salmonella* infection.

Human challenge studies involve the administration of *S*. Typhi to consenting healthy adult volunteers with the intent to deliberately induce infection under carefully controlled conditions. Such models are safe and provide a cost effective method to expedite vaccine development and facilitate the discovery and assessment of novel diagnostics. In comparison to large field trials designed to demonstrate vaccine efficacy, challenge models are more cost effective, less labor intensive and can be completed within a shorter study period. Typhoid challenge studies using a relevant host can be extremely informative and have historically contributed to knowledge and understanding of strain virulence, infectious dose, microbial pathogenesis, immunity and the identification of potential vaccine candidates (Hornick et al., 1970a,b, 2007). The re-establishment in Oxford of a controlled human infection model has provided a rare opportunity to make direct comparisons to the findings from historical human studies by using the same challenge strain (Quailes) (Waddington et al., 2014b). The models provide clues to possible correlates of protection, and allow direct comparisons between individuals who do or do not succumb to infection. The collection of baseline (pre-exposure) samples for assessment in combination with pre/post-vaccination and subsequent challenge is a remarkable opportunity. The ability to control the timing of infection in a well-defined study cohort is of unique value and an important research approach by which to understand disease pathogenesis, now greatly enhanced by advances in scientific areas such as functional genomics and systems biology approaches. In addition, identification of host genetic factors linked to infection susceptibility will supplement our knowledge as genetic variation within distinct populations has been linked to defense against typhoid fever (Ziakas et al., 2013).

Despite providing the opportunity for study of *S*. Typhi infection in the natural host, human challenge studies are associated with known limitations. Often only a short-term duration of infection can be studied due to early treatment and patient safety, while the criteria for diagnosis of typhoid fever in the model may differ from that used in an endemic setting. Specific details of the challenge model (including the choice of microbial strain, method of administration and the challenge dose) may not extrapolate to the natural course or mode of infection. Challenge volunteers may not represent the final target population of the vaccine and observations may not always be transferable to an endemic population due to inherent differences in nutrition, host microbiome, or the absence of co-infection with other intestinal bacteria or parasites (Ahmer and Gunn, 2011; Hallstrom and McCormick, 2011; Nuccio and Bäumler, 2014). As the safety of consenting individuals is of the upmost importance, the design of such studies must be ethically sound, ensuring minimal risk to participants while maintaining scientific integrity (Miller and Grady, 2001; Hope and McMillan, 2004).

## Development of improved diagnostics

Diagnosis of enteric fever in an endemic setting is still heavily reliant on clinical presentation and notoriously unreliable tests reiterate the poor sensitivity and specificity of current diagnostics (Parry et al., 2011). The next generation diagnostic point of care test for *S*. Typhi must be rapid, accurate and affordable in the setting in which it is ultimately destined, and ideally will not rely on expensive equipment, nor highly skilled and trained clinical and laboratory personnel (Andrews et al., 2013). The search for an improved gold standard typhoid fever diagnostic remains a challenge for all researchers, but one approach is to use samples collected during challenge models as an alternative to those from endemic-settings (Baker et al., 2010; Nga et al., 2010). The model provides a defined time point at which infection takes place and allows the collection of samples before, during and after the onset of clinical disease. Longitudinal challenge model samples (including serum, plasma, saliva, urine, stool and peripheral blood lymphocytes) collected in Oxford will be used to support, validate or indeed refute the accuracy and suitability of a novel diagnostic test.

Recent advances in *Salmonella* diagnostics include metabolomics for the detection of *Salmonella* in blood during infection. Relying on the identification of a unique metabolomic signature in an infected individual, this technique has the potential to discriminate between *S*. Typhi and *S*. Paratyphi A infection (Näsström et al., 2014). Antibody microarrays are being used to identify potential biomarkers of infection for application in the development of novel diagnostic tests and subunit vaccine targets (Liang et al., 2013; Bhuiyan et al., 2014). These approaches are being applied to the challenge model to validate their use at different stages of infection in a naive population. Simple antibody profiles in a small blood sample collected upon hospital presentation have been proposed as an achievable typhoid/paratyphoid fever diagnostic (TPTest) in an endemic setting equipped with a basic microbiology laboratory (Khanam et al., 2013). Using only a few micro liters of a clinical specimen, it is now possible to employ high throughput screening techniques to identify early signatures of the host response, thereby increasing the possibility of a future diagnostic based on a non-invasive sample type such as saliva or urine (Zaka-ur-Rab et al., 2012; Das et al., 2013). Challenge studies provide a unique myriad of resources which through discussion and development with international collaborators has the potential to improve both the quality and standardization of future *Salmonella* diagnostics.

## Concluding thoughts

The knowledge gained from human challenge models will not only identify the elusive correlate of protection, but will inform and facilitate the accelerated development of novel diagnostics, identification of novel vaccine candidates, and the targeted assessment of next generation bivalent or combination vaccines. Human challenge models will continue to be instrumental in unraveling the complex pathways associated with infection and will help address remaining questions about *S*. Typhi pathogenesis. We acknowledge the volunteers who participate in *Salmonella* challenge trials and help in the quest to conquer enteric fever.

### Conflict of interest statement

The reviewer Calman A. MacLennan declares that, despite having collaborated with author Andrew J. Pollard, the review process was handled objectively and no conflict of interest exists. The Guest associate editor Constantino López-Macías declares that, despite holding an honorary position at the same institution as the authors, the review process was handled objectively and no conflict of interest exists. The authors declare that the research was conducted in the absence of any commercial or financial relationships that could be construed as a potential conflict of interest.
